# Supramolecularly Engineered Conjugate of Bacteria and Cell Membrane‐Coated Magnetic Nanoparticles for Enhanced Ferroptosis and Immunotherapy of Tumors

**DOI:** 10.1002/advs.202304407

**Published:** 2023-10-18

**Authors:** Beibei Xie, Huichao Zhao, Yuan‐Fu Ding, Ziyi Wang, Cheng Gao, Shengke Li, Kehan Zhang, Seong Wan Ip, Huazhong Yu, Ruibing Wang

**Affiliations:** ^1^ State Key Laboratory of Quality Research in Chinese Medicine Institute of Chinese Medical Sciences University of Macau Taipa Macau 999078 China; ^2^ MoE Frontiers Science Center for Precision Oncology University of Macau Taipa Macau 999078 China; ^3^ Macau Anglican College Taipa Macau 999078 China; ^4^ School of the Nations Taipa Macau 999078 China; ^5^ Department of Chemistry and Department of Molecular Biology and Biochemistry Simon Fraser University British Columbia Burnaby V5A 1S6 Canada

**Keywords:** cell membranes, engineered bacteria, ferroptosis, supramolecular

## Abstract

Although various ferroptosis inducers including magnetic nanoparticles (Fe_3_O_4_) and iron‐organic frameworks have been applied in cancer treatment, the mild immunogenicity, low targeting efficiency to the tumor, and poor tissue penetration have limited the therapeutic efficacy. Herein, a supramolecularly engineered conjugate between living bacteria (facultative anaerobic *Salmonella typhimurium* VNP20009, VNP) and cancer cell membranes‐coated Fe_3_O_4_ nanoparticles is developed for improving targeted delivery of Fe_3_O_4_ nanoparticles into the tumor tissue and for synergistic ferroptosis and immunotherapy of tumor. The enhanced ferroptosis induced by both Fe_3_O_4_ nanoparticles and the loaded ferroptosis inducing agent (sulfasalazine (SAS)) effectively inhibits tumor growth and generates immune response via immunogenic cell death (ICD). The colonization of VNP in tumors also induces adaptive immune responses and further promotes ferroptosis. Fundamentally, the supramolecular conjugate of VNP and cell membranes‐coated Fe_3_O_4_ can potentiate the therapeutic capability of each other through mutually magnifying the ferroptosis and immunotherapy, resulting in significantly enhanced antitumor effects.

## Graphical abstract

A supramolecularly engineered conjugate between living bacteria and cancer cell membranes‐coated Fe_3_O_4_ nanoparticles is developed for improving targeted delivery of Fe_3_O_4_ nanoparticles into the tumor tissue for synergistc ferroptosis and immunotherapy of tumor.

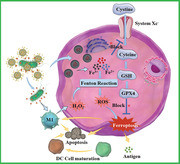

## 1 Introduction

Ferroptosis is a newly discovered iron‐dependent cell death pathway.^[^ ^1^, ^2^ ^]^ The glutathione (GSH)‐dependent excessive production and accumulation of reactive oxygen species (ROS) induced by the iron‐based Fenton reaction initiates ferroptosis,^[^ ^3^ ^]^ which could effectively inhibit tumor growth and generate mild immunogenicity.^[^ ^4^, ^5^ ^]^ Considering the central role of iron in ferroptosis, various ferroptosis inducers such as ferumoxytol,^[^ ^6^ ^]^ magnetic nanoparticles (Fe_3_O_4_),^[^ ^7^, ^8^ ^]^ iron‐organic frameworks^[^ ^9^ ^]^ and ferrocene^[^ ^10^ ^]^ have been employed for ferroptosis‐related cancer treatment. However, the mild immunogenicity, low tumor targeting, and poor tissue penetration have limited the overall therapeutic efficacy.

On the one hand, in recent years, biomimetic nanomedicine based on cell membranes coating technology have demonstrated considerable promises for biomedical applications, particularly the targeted drug delivery.^[^ ^11^, ^12^ ^]^ Cancer cell membranes expressing “markers of self” and “self‐recognition molecules” could wrap around the surface of nanoparticles via this coating technique and bestow the natural ability of targeting the homotypic tumor cells to the nanoparticles.^[^ ^13^, ^14^ ^]^ Compared with uncoated nanoparticles, cancer cell membranes‐coated nanoparticles could significantly increase the stability of nanoparticles in physiological conditions and enhance the accumulation of nanoparticles in tumors via homing effects.^[^ ^15^, ^16^ ^]^ The unique bio‐interfacing capabilities enable the cancer cell membranes‐capped nanoparticles as targeted carriers for nanomedicines delivery.

On the other hand, tumors especially the solid tumors are well known to exhibit intra‐tumor anaerobic conditions, eutrophication, and immunosuppressive microenvironment, due to the poorly formed vasculature system and the rapid proliferation of tumor cells.^[^ ^17^, ^18^, ^19^ ^]^ The unique microenvironment of tumors serves as an ideal habitat for various anaerobic and facultative anaerobes.^[^ ^20^, ^21^, ^22^, ^23^, ^24^, ^25^ ^]^ Meanwhile, bacteria as immune modulators and drug delivery vehicles have attracted increasing attention in tumor biotherapy.^[^ ^26^, ^27^, ^28^ ^]^ Colonization of bacteria in tumor tissues may induce innate and adaptive immune responses against the tumor cells, achieving nonspecific killing of heterogeneous tumor cells.^[^ ^29^, ^30^ ^]^ Consequently, the use of bacteria as delivery vehicles can improve the accumulation and penetration of medicines in tumors and hence improve their therapeutic efficacies.

**Figure 1:**
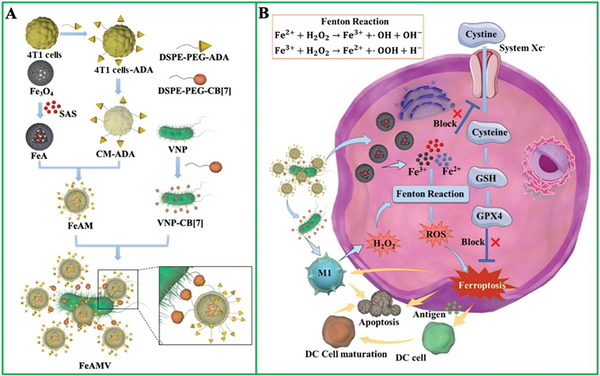
The construction of a supramolecular conjugate of living bacteria and cell membranes‐coated ferroptosis inducer. A) Scheme showing the preparation of conjugate of CM‐ADA coated Fe_3_O_4_ and CB[7] modified VNP via host–guest interactions. B) The specific accumulation of FeAM at the tumor sites and subsequent ferroptosis and immunotherapy against tumors.

Herein, we develop a host–guest chemistry mediated supramolecular conjugate of living bacteria and cell membranes‐coated Fe_3_O_4_ nanoparticles for the synergistic ferroptosis and immunotherapy against tumors with enhanced specificity and efficacy. As shown in **Figure** **1**A, facultative anaerobic *Salmonella typhimurium* VNP20009 (VNP) with excellent safety, anticancer activity and tumor‐targeting capability ^[^ ^21^, ^31^, ^32^ ^]^ were selected as living delivery carriers. The supramolecular host precursor (cucurbit[7]uril, CB[7]) and guest precursor (adamantine, ADA) are conjugated onto the surface of VNP and cancer cell (4T1) via simple lipid ligand membrane‐insertion, respectively. A ferroptosis agent, sulfasalazine (SAS), is loaded into porous Fe_3_O_4_ nanoparticles (FeA). Subsequently, 4T1 cell membranes (CM) inserted with ADA (CM‐ADA) are coated on the surface of FeA to encapsulate SAS (namely, FeAM). The supramolecular conjugate of FeAM and CB[7] modified VNP (VNP‐CB[7]), abbreviated as FeAMV, is obtained via strong host–guest interaction between CB[7] and ADA (Ka > 10^6^ m ^−1^) for VNP‐hitchhiking drug delivery. As shown in Figure 1B, after intravenous injection, FeAMV specifically accumulates at the tumor sites, due to the excellent tumor‐tropism of VNP and homing effects of CM, respectively. Once FeAMV is taken up by tumor cells, CM would be destroyed during the transmembrane transportation, and FeA is exposed. The released SAS from FeA restrains the uptake of cysteine through inhibiting the pathway of glutathione peroxidase 4 (GPX4)‐cystine antiporter system Xc‐transporter (XcT) that would otherwise allow cystine to enter the cells for synthesis of GSH, leading to promoted ferroptosis.^[^ ^33^ ^]^ As a ferroptosis inducer, Fe_3_O_4_ nanoparticles work together with SAS to synergistically initiate ferroptosis through Fenton reaction, inhibiting tumor growth. The enhanced ferroptosis also significantly improves maturation of dendritic cells (DCs) and M1 polarization of macrophages in tumor tissues, driving immunotherapy. Moreover, the colonization of VNP in tumor sites also promotes M1 polarization, which not only improves the intracellular H_2_O_2_ level,^[^ ^34^ ^]^ but also induces the adaptive immune responses, promoting the ferroptosis and immunotherapeutic efficacy of tumors. Hence, the supramolecular conjugate of VNP and Fe_3_O_4_ nanoparticles potentiates the therapeutic capability of each other through mutually magnifying the ferroptosis and immunotherapy, resulting in the effective therapy of tumors.

## 2 Results and Discussion

### 2.1 Preparation and Characterization

**Figure 2:**
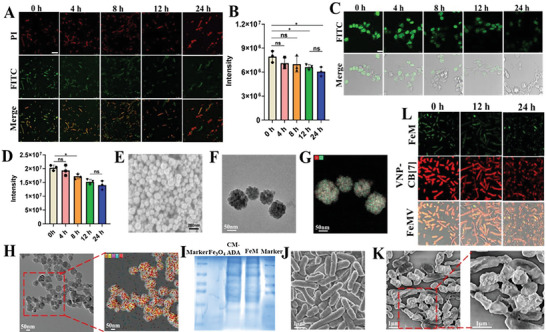
Preparation and characterization of VNP‐CB[7], CM‐ADA, FeM, and FeMV. A) VNP‐CB[7] were incubated with PBS containing FITC‐ADA and observed by CLSM after further incubation with fresh blank PBS for 4, 8, 12, and 24 h. VNP was stained by PI with red fluorescence, and CB[7] on surface of VNP was stained by FITC with green fluorescence. The scale bar was 5 µm. B) Quantitative analysis of the fluorescence intensities of FITC. C) ADA decorated 4T1 cells were incubated with blank DMEM containing 10 µm CD‐FITC and observed by CLSM after further incubation with fresh blank DMEM for 4, 8, 12, and 24 h. The scale bar was 5 µm. D) Quantitative fluorescence intensities analysis of FITC. E) SEM, F) TEM image and G) the elemental mapping images of Fe and O element of Fe_3_O_4_ nanoparticles. H) TEM image and the elemental mapping images of C, N, O, P and Fe element of FeM. I) The protein bands of marker, Fe_3_O_4_, CM‐ADA and FeM in the gel by Coomassie Blue staining. The SEM image of J) VNP and K) FeMV. L) CLSM images of FeMV were incubated with fresh blank DMEM for different time (FeM labeled with FITC and VNP‐CB[7] labeled with PI). B) and D): Results were based on three independent experiments (*n* = 3) and presented as mean ± standard deviation (SD), statistical significance was evaluated using an un‐paired two‐tailed *t*‐test (^*^ *p* < 0.05, ^**^ *p* < 0.01, ^***^ *p* < 0.001, ^****^ *p* < 0.0001).

In order to decorate the surface of VNP with CB[7], a lipid molecule,1,2‐distearoyl‐sn‐glycero‐3‐phosphoethanolamine‐poly(ethyleneglycol) (DSPE‐PEG),^[^ ^35^, ^36^, ^37^, ^38^ ^]^ was conjugated to CB[7] via a thiolene click reaction between DSPE‐PEG‐SH and monoallyloxy CB[7] (Figure S1, Supporting Information) ^[^ ^39^ ^]^ As shown in Figure S2 (Supporting Information), ^1^H NMR spectra confirmed the successful conjugation of CB[7] with DSPE‐PEG (DSPE‐PEG‐CB[7]). Subsequently, VNP was incubated with DSPE‐PEG‐CB[7] to obtain CB[7] modified VNP, VNP‐CB[7], through membrane insertion. The ferrocene was incubated with VNP‐CB[7] through the strong CB[7]‐ferrocene host–guest interactions to measure the quantity of CB[7] on VNP through calculating iron content, suggesting ≈4 nmol of CB[7] per 10^7^ CFU of VNP. To evaluate the stability of membrane decoration of CB[7] in VNP, fluorescein isothiocyanate (FITC) modified ADA (FITC‐ADA) was used for labeling CB[7] on VNP through host–guest interactions between CB[7] and ADA. As shown in **Figure** **2**A, a bright green fluorescence of FITC was observed after incubation for 4 h and still maintained a high intensity of fluorescence for 24 h post‐incubation (Figure 2B), indicating the high stability of membrane decoration of CB[7] on VNP, suitable for in vivo delivery.

In order to decorate the surface of CM with ADA, DSPE‐PEG‐ADA was firstly used to insert the membrane of 4T1 cells according to our previously published method.^[^ ^40^ ^]^ The FITC modified *β*‐cyclodextrin (FITC‐CD) was utilized for labeling ADA on 4T1 cells to study the stability of membrane decoration. 4T1 cells inserted with ADA were incubated with FITC‐CD for 2 min, a bright green fluorescence was observed in 4T1 cells (Figure 2C) and still maintained a high intensity of fluorescence for 4 h post‐incubation (Figure 2D). After post‐incubation for 12 h (Figure 2C,D), the green fluorescence intensity in 4T1 cells decreased gradually due to the cell membrane's fluidity and the phagocytosis of 4T1 cells.^[^ ^41^ ^]^ Hence, to obtain the CM‐ADA with reasonable stability and a large loading amount of ADA, membrane of 4T1 cells was immediately collected from harvested cells after incubation with DSPE‐PEG‐ADA for 1.5 h.

Porous Fe_3_O_4_ nanoparticles were synthesized refer to a previously reported method.^[^ ^42^ ^]^ Both SEM (Figure 2E) and TEM images (Figure 2F,G) indicated that Fe_3_O_4_ nanoparticles exhibited a porous structure with an average size of 80 nm (Figure S3A, Supporting Information). The cancer cell membranes could potentially bestow nanoparticles the natural ability of targeting the homotypic tumor cells^[^ ^12^ ^]^ and increase the stability of nanoparticles in physiological conditions.^[^ ^11^ ^]^ In order to enable Fe_3_O_4_ nanoparticles to have the targeting ability and conjunct with VNP, the CM‐ADA was used to coat the surface of Fe_3_O_4_ nanoparticles by an ultrasonic method to obtain cancer cell‐mimetic Fe_3_O_4_ (FeM). The average size increased from 80 nm of Fe_3_O_4_ to 95 nm of FeM after coating of CM‐ADA (Figure S3B, Supporting Information), indicating the thickness of the CM‐ADA layer coated on Fe_3_O_4_ was ≈15 nm. After 7 days, the very modest size variation of FeM confirmed the good hydrodynamic stability of FeM (Figure S3B, Supporting Information). The smaller size variation of FeM than that of Fe_3_O_4_ (Figure S3A, Supporting Information) indicated that the coating of CM improved the stability of Fe_3_O_4_. Serum protein adsorption was evaluated to investigate the stealth property of nanoparticles, by measuring the hydrodynamic diameter changes of the nanoparticles before and after incubation with fetal calf serum (FBS). The smaller size variation of FeM than that of Fe_3_O_4_ nanoparticles indicated that the coating of CM could reduce nonspecific protein adsorption and increase the stability of Fe_3_O_4_ nanoparticles (Figure S4, Supporting Information). The coexistence and uniform distribution of C, N, O, P, and Fe element in EDS spectra of FeM further confirmed the successful coating of CM‐ADA on the surface of Fe_3_O_4_ (Figure 2H). The protein bands of FeM in the gel by Coomassie Blue staining were similar to those of CM‐ADA (Figure 2I), suggesting successful coating Fe_3_O_4_ by CM‐ADA. The decreased Zeta potential also confirmed the conjugation of CM‐ADA onto the surface of Fe_3_O_4_ (Figure S5, Supporting Information).

FeM was conjugated with VNP‐CB[7] to form FeMV through strong CB[7]‐ADA host–guest interactions. Compared with the clear and smooth surface of VNP (Figure 2J), the Fe_3_O_4_ nanoparticles homogeneously dispersed on the surface of FeMV and hence exhibited the rough surface of FeMV (Figure 2K). The loading amount of Fe_3_O_4_ nanoparticles was calculated to be ≈10^−5^ nmol per 10^7^ CFU of VNP through weight difference method. To study the stability of FeM on VNP‐CB[7], Fe_3_O_4_ nanoparticles were loaded with FITC. PI with red fluorescence was used to label VNP‐CB[7]. As shown in Figure 1L, the co‐existence of bright red fluorescence of PI and green fluorescence of FITC after incubation for 24 h indicated good stability of the supramolecular conjugate of live bacteria and cell‐mimetic Fe_3_O_4_, which is suitable for in vivo delivery. The similar proliferation profiles of VNP and FeMV indicated that the conjugation of FeM had negligible effect on the proliferation of VNP (Figure S6, Supporting Information).

### 2.2 SAS Loading and Release

**Figure 3:**
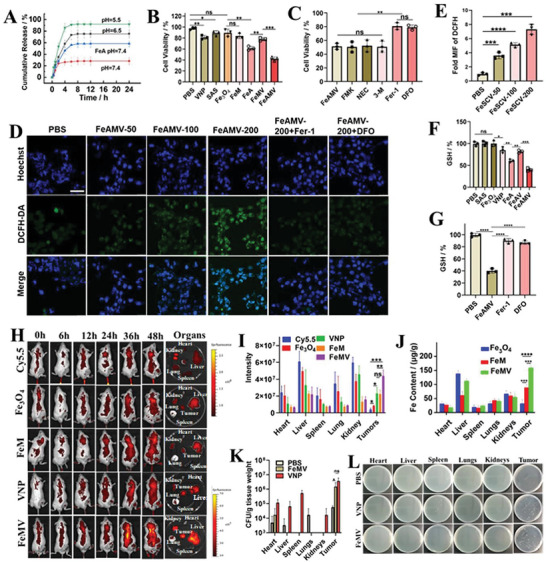
In vitro cytotoxicity and mechanism of ferroptosis induced by FeAMV as well as the in vivo bio‐distribution of various formulations. A) Release profiles of SAS in FeA and FeAM in PBS solutions with different pH (pH = 7.4, 6.5, and 5.5). B) The viability of 4T1 cells treated with various formulations containing the equivalent concentration of SAS (20 µg mL^−1^), Fe_3_O_4_ (100 µg mL^−1^) and VNP (10^6^ CFU) after 24 h incubation. C) The viability of 4T1 cells treated with FeAMV after addition with PBS, FMIK, NEC, 3‐M, Fer‐1, and DFO, respectively. D) The quantitative fluorescence intensities of DCFH‐DA inconfocal images of 4T1 cells incubated with FeAMV of different concentrations and FeAMV with addition of Fer‐1 and DFO, respectively. E) The corresponding fluorescence intensities of DCFH‐DA in 4T1 cells incubated with FeAMV of different concentrations. The scale bar was 25 µm. F) The GSH content of 4T1 cells treated with different formulations. G) The GSH content of 4T1 cells treated with PBS, FeAMV, FeAMV+Fer‐1, FeAMV+DFO, respectively. H) In vivo fluorescence imaging of the tumor‐bearing mice and *ex vivo* imaging of harvested the main organs including heart, liver, spleen, lung, kidney, and tumor after intravenous injection with different formulations loaded with the equivalent amount of Cy5.5, respectively. The quantitative analysis of I) Cy5.5, J) Fe, K) VNP and L) the corresponding photographs of VNP in the heart, liver, spleen, lungs, kidneys, and tumor harvested from the tumor‐bearing mice after intravenous injection for 48 h. A), B), C), E), F), G), I), J) and K): Results were based on three independent experiments (*n* = 3) and presented as mean ± SD, statistical significance was evaluated using an un‐paired two‐tailed *t*‐test (^*^ *p* < 0.05, ^**^ *p* < 0.01, ^***^ *p* < 0.001, ^****^ *p* < 0.0001).

Benefiting from a large specific surface of 136 m^2^ g^−1^ (Figure S7A, Supporting Information) and porous structures with a mean pore size of 3.8 nm (Figure S7B, Supporting Information) of Fe_3_O_4_ nanoparticles, the loading ratio of SAS in Fe_3_O_4_ nanoparticles was as high as 20.1% and the encapsulation efficiency was 79.1%. The loading amount and encapsulation efficiency of SAS in FeAM were 19.5% and 78.5%, respectively, very close to those of FeA, suggesting less loss of SAS due to the CM‐ADA coating. The in vitro release profile of SAS from FeAM under different pH conditions was evaluated (**Figure** **3**A). FeAM exhibited a slower SAS release kinetics compared with that of FeA under physiological conditions (pH = 7.4), indicating that the coating of CM‐ADA protected SAS from nonspecific release. In contrast with the less release of SAS at pH of 7.4, ≈70% of SAS was released from FeAM at pH 6.5, while 91% of SAS was released from FeAM at pH 5.5, indicating the acid‐responsive release of SAS from FeAM.

### 2.3 In Vitro Cytotoxicity and Mechanism of Ferroptosis

The carrier of Fe_3_O_4_ nanoparticles, FeM and FeMV showed very mild cytotoxicity against LO2 cells at the concentrations of 100 µg mL^−1^ (Figure S8, Supporting Information), indicating their excellent biocompatibility. We further evaluated the cytotoxicity of various formulations containing the equivalent concentration of SAS, Fe_3_O_4_ and VNP against 4T1 cells (Figure 3B). As reported that VNP has anti‐cancer activity,^[^ ^21^ ^]^ in our study VNP reduced cell viability of 4T1 cells (Figure 3B). FeA exhibited stronger cytotoxicity against 4T1 cells than that of free SAS and Fe_3_O_4_, due to that the combination of SAS and Fe_3_O_4_ might synergistically enhance ferroptosis. Compared with Fe_3_O_4_, FeM exhibited improved cytotoxicity against 4T1 cells due to the increased cellular uptake of nanoparticles upon coating of CM. As expected, FeAMV exhibited the best anti‐cancer effect among all the studied groups.

To explore the mechanism of FeAMV‐induced cytotoxicity against 4T1 cells, apoptosis inhibitor (FMK), necroptosis inhibitor (Necrostatin‐1, NEC) and autophagy inhibitor 3‐methladenine (3‐MA) were utilized to analyze the possible death pathways. As shown in Figure 3C, the respective addition of FMK, NEC, and 3‐MA had almost no effects on the viability of FeAMV‐treated 4T1 cells, indicating that FeAMV did not induce the occurrence of apoptosis, necroptosis or autophagy in 4T1 cells. Moreover, the effect of ferroptosis inhibitor ferrostatin‐1 (Fer‐1) and iron chelator deferoxamine (DFO) on the viability of FeAMV‐treated 4T1 cells was respectively examined. It was found that both Fer‐1 and DFO could significantly increase viability of FeAMV‐treated 4T1 cells (Figure 3C), suggesting that the cell death here was driven by ferroptosis.

Considering that the ferroptosis induced by the Fenton reaction could generate ROS, the effects of FeAMV on the level of ROS in 4T1 cells was evaluated through a 2,7‐dichlorodi‐hydrofluorescein diacetate (DCFH‐DA) probe.^[^ ^8^ ^]^ Compared with PBS groups, after treatment with FeAMV, the cellular ROS level of 4T1 cells was significantly elevated in a dose‐dependent manner (Figure 2D,E), while the intracellular ROS level in FeAMV‐treated cells was significantly downregulated by addition of Fer‐1 or DFO (Figure 2D,E), indicating ferroptosis occurred in FeAMV‐treated 4T1 cells.

The influence of FeAMV on GSH level in 4T1 cells was also measured by GSH quantitative assay kit to further verify the GSH‐relevant ferroptosis (Figure 3F). Compared with free SAS and Fe_3_O_4_, the FeA could reduce the GSH level due to the enhanced ferroptosis induced by the combination of SAS and Fe_3_O_4_. VNP could also reduce the GSH level, which indicated that VNP could promote ferroptosis. Compared with other groups, FeAMV induced the lowest cellular GSH level, and the cellular GSH level was significantly increased with the addition of both Fer‐1 and DFO (Figure 3G), which further confirmed the ferroptosis occurrance in FeAMV‐treated cells.

### 2.4 In Vivo Bio‐Distribution

The bio‐distribution of Fe_3_O_4_, VNP, FeM, and FeMV loaded with the equivalent amount of Cy5.5 was respectively investigated in 4T1 tumor‐bearing mice model. To label the VNP with Cy5.5, the ADA modified liposome loaded with Cy5.5 was inserted on the membrane of VNP‐CB[7] through the host–guest interaction between CB[7] and ADA. In the free Cy5.5 group, a large amount of Cy5.5 quickly accumulated in the liver, spleen, and kidney (Figure 3H), but less fluorescence of Cy5.5 was observed in tumors (Figure 3I). The highest fluorescence intensity of Cy5.5 in liver was observed among all the organs of Fe_3_O_4_ group, indicating Fe_3_O_4_ nanoparticles were mainly metabolized by the liver (Figure 3H,I). Compared with the mice treated with free Cy5.5, bright red fluorescence of Cy5.5 was observed in the tumor sites of mice treated with Fe_3_O_4_, due to the enhanced permeability and retention induced by Fe_3_O_4_ carriers. In contrast with mice treated with Fe_3_O_4_, the mice treated with FeM exhibited higher fluorescence of Cy5.5 and Fe content (Figure 3J) in tumors, likely due to the enhanced encapsulation, permeability and targeting ability mediated by CM. The higher fluorescence intensity of Cy5.5 in spleen than other organs in VNP group indicated VNP was mainly metabolized by the spleen (Figure 3J,K). Benefiting from the unique property of hypoxia targeting, VNP exhibited a large fraction of accumulation in tumors (Figure 3J,K), confirming the high tumor‐targeting efficiency of VNP. Among all groups, the highest fluorescence intensity of Cy5.5, the largest amount of Fe and VNP were observed in the tumor of mice treated with FeMV, which confirmed the excellent targeting ability of the formulation due to the presence of both VNP and CM (Figure 3K).

### 2.5 In Vitro Immune Activation Induced by FeAMV‐Mediated Ferroptosis

**Figure 4:**
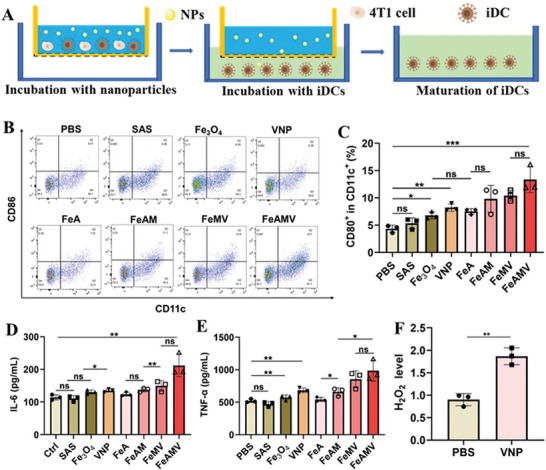
In vitro immune response induced by FeAMV‐mediated ferroptosis. A) The scheme of the co‐culture system of 4T1 cells and bone marrow‐derived iDCs. B) The flow cytometric analysis and C) quantification of mature DCs (CD86^+^/CD11c^+^, gated on CD11c^+^ cells) after incubation with various formulations for 24 h. D) TNF‐*α* and E) IL‐6 expression level in Raw264.7 cells incubated with different groups for 24 h. F) The generated H_2_O_2_ level in Raw 264.7 cells after incubation with VNP (10^6^ CFU) for 48 h. C), D), E) and F): Results were based on three independent experiments (*n* = 3) and presented as mean ± SD, statistical significance was evaluated using an un‐paired two‐tailed *t*‐test (^*^ *p* < 0.05, ^**^ *p* < 0.01, ^***^ *p* < 0.001, ^****^ *p* < 0.0001).

The immune activation in vitro induced by FeAMV‐mediated ferroptosis was evaluated. As the antigen‐presenting cells, DCs are involved in the maintenance of T‐cell‐mediated immune response.^[^ ^43^ ^]^ The antigens could promote the maturation of immature DCs (iDCs), which could produce peptide‐major histocompatibility complex molecules to T cells, generating effector T cells to destroy tumors.^[^ ^44^ ^]^ Hence, the level of maturation of DCs was evaluated as a sign of the immune activation response. As shown in **Figure** **4**A, 4T1 cells were firstly cultured in the upper wells (donor wells) with various formulations, and then iDCs were seeded in the bottom wells (receptor wells) were co‐cultured with the upper 4T1 cells for another 24 h. Subsequently, the DCs were collected and subjected to flow cytometry to analyze the expressions of DCs maturation markers, costimulatory molecules CD86 and CD11c. Compared with the group of free Fe_3_O_4_ and SAS, the ratio of CD86^+^ in CD11c^+^ in both FeA and VNP group exhibited significant increase (Figure 4B,C), indicating both ferroptosis and VNP could induce the immune activation and trigger the effective maturation of DCs. Benefiting from the synergistically enhanced immune activation induced by ferroptosis and VNP, the group of FeAMV possessed the largest ratio of CD86^+^ in CD11c^+^ (Figure 4C).

As the DC activation‐related cytokines, the TNF‐*α* and interleukin 6 (IL‐6) were also evaluated to further confirm immune activation.^[^ ^45^ ^]^ Raw264.7 cells co‐cultured with FeAMV produced the largest amount of IL‐6 (Figure 4D) and TNF‐*α* (Figure 4E) among all the groups, consistent with the level of DC maturation. Moreover, the immune response induced by VNP could also improve the generation of ROS (Figure S9, Supporting Information), especially H_2_O_2_ (Figure 4F), the resultant high intracellular level of H_2_O_2_ could initiate Fenton reaction and hence promote the ferroptosis. These above results indicated that FeAMV‐mediated tumor cell ferroptosis significantly improved the cellular immune response.

### 2.6 In Vivo Anti‐Tumor Therapy

**Figure 5:**
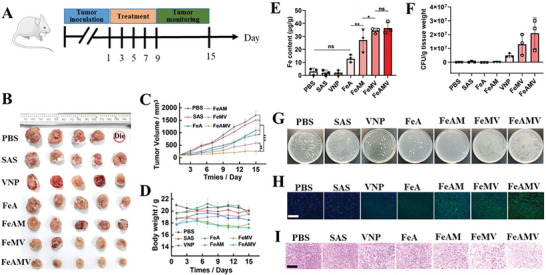
In vivo anti‐tumor efficacy of FeAMV, via ferroptosis and immunotherapy. A) The animal study protocol. B) The digital tumor images, C) tumor growth profiles and D) body weight changes of 4T1 tumor‐bearing mice during the treatment with PBS, SAS, VNP, FeA, FeAM, FeMV, and FeAMV, respectively, with an equivalent SAS dose of 5 mg k^−1^g. E) Fe content, F) VNP content and G) the corresponding photographs of VNP in tumor tissues harvested from all the groups after treatment. Histological observation of the harvested tumor tissues stained with H) TUNEL and I) H&E. Scale bar: 50 µm. C) and D): Results were based on five independent experiments (*n* = 5) and presented as mean ± S.D., E) and F): Results were based on three independent experiments (*n* = 3) and presented as mean ± S.D., statistical significance was evaluated using an un‐paired two‐tailed *t*‐test (^*^ *p* < 0.05, ^**^ *p* < 0.01, ^***^ *p* < 0.001, ^****^ *p* < 0.0001).

The antitumor effect of FeAMV in vivo was evaluated in 4T1‐tumor‐bearing mice. The tumor‐bearing mice were randomly divided into seven groups (*n* = 5 in each group), and various formulations were respectively injected into the mice on Day 1, 3, 5, 7, and 9 through the tail veins (**Figure** **5**A). As shown in the final tumor images (Figure 5B) and tumor volume evoling profile (Figure 5C), the tumors of PBS treated group grew most rapidly. Similarly, the free SAS group also exhibited negligible antitumor efficacy. VNP exhibited modest anti‐tumor effect, due to bacteria‐induced innate and adaptive immune responses.^[^ ^21^ ^]^ In comparison with the free SAS group, the FeA group exhibited obvious antitumor effects, due to the enhanced ferroptosis induced by both SAS and Fe_3_O_4_. FeAM group showed higher anti‐proliferative effects than that of FeA group due to the encapsulation, permeability and targeting ability induced by CM. Attributed to the enhanced ferroptosis and immune response of ferroptosis inducers (Fe_3_O_4_ and SAS) and VNP, FeAMV group showed the highest anti‐proliferative effects among all the groups. In addition, the moderate change in the body weight of FeAMV group indicated the low toxicity of FeAMV (Figure 5D). Benefiting from the excellent targeting ability of both CM and VNP to the tumors, the largest amount of Fe content (Figure 5E) and VNP amount (Figure 5F,G) were observed in the tumors of mice from FeAMV group. Both the results of immunofluorescence TUNEL (Figure 5H) and H&E (Figure 5I) analysis of the tumors confirmed that FeAMV exhibited the highest antitumor effects and most tumor cell apoptosis among all the tested groups. The above results confirmed that FeAMV could synergistically magnify ferroptosis and immunotherapy induced by both VNP and Fe_3_O_4_, resulting in the effective therapy of tumors.

### 2.7 FeAMV‐Mediated Ferroptosis for In Vivo Immune System Activation

**Figure 6:**
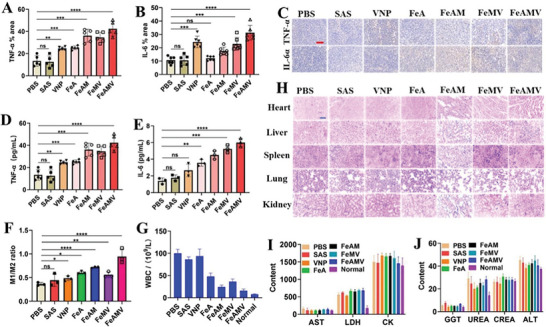
FeAMV‐mediated ferroptosis for in vivo immune response and in vivo safety evaluation. A) TNF‐*α* and B) IL‐6 analysis of tumor tissues determined by flow cytometry and C) the corresponding immunostaining of tumor tissues collected from the treated mice with different formulations. The analysis of D) TNF‐*α* and E) IL‐6 from the blood samples collected from the treated mice with different formulations. F) The ratio of M1/M2 of tumor tissues determined by analysis the ratio of CD11c (an M1 marker) CD206 (an M2 marker). The analysis of G) WBC from the blood samples collected from the normal mice and treated mice. H) Histopathological examination (HE) of the harvested heart, liver, spleen, lungs, and kidneys from the different groups. The analysis of I) AST, LDH, and CK, and J) GGT, UREA, CREA, and ALT from the blood samples collected from the normal mice and treated mice. Scale bar: 50 µm. A), B) and D) Results were based on five independent experiments (*n* = 5) and presented as mean ± SD, E), F) and G): Results were based on three independent experiments (*n* = 3) and presented as mean ± SD, statistical significance was evaluated using an un‐paired two‐tailed *t*‐test (^*^ *p* < 0.05, ^**^ *p* < 0.01, ^***^ *p* < 0.001, ^****^ *p* < 0.001).

To evaluate the in vivo immune response induced by the treatment with various formulations, the expression level of representative immune cytokines TNF‐*α* and IL‐6 in both tumors and serum were examined. There was nearly negligible overexpression of TNF‐*α* (**Figure** **6**A) and IL‐6 (Figure 6B) from the mice of the PBS and free SAS group, while FeA could promote the expression of TNF‐*α* and IL‐6, due to ferroptosis. FeAM group exhibited higher expression of TNF‐*α* and IL‐6 than that of FeA group, due to the enhanced permeability and targeting ability induced by CM. VNP with unique property of hypoxia targeting could also induce innate and adaptive immune responses in tumors, resulting in the upregulated expression of TNF‐*α* and IL‐6. Both the immunostaining (Figure 6C) and immune cytokines analysis (Figure 6D and 6E) of tumor tissues confirmed that FeAMV‐mediated ferroptosis exhibited the highest immune response among all the tested groups, due to the synergistically enhanced immune activation induced by both ferroptosis and VNP. Considering that the immune response could promote the polarization of anti‐inflammatory macrophages (M2) to pro‐inflammatory macrophages (M1). Hence, the ratio of M1/M2 determined by analysis of the ratio of CD11c (an M1 marker) and CD206 (an M2 marker) was evaluated to further analyze the immune system activation. It was found that the FeAMV‐treated mice exhibited the largest ratio of M1/M2 (Figure 6F) among all the tested groups, suggesting that FeAMV‐mediated tumor cell ferroptosis significantly improved the immune response in vivo. Moreover, the colonization of VNP in tumor tissues may induce the mild infections, resulting in an increased number of white blood cells (WBC) than that of normal mice (Figure 6G).

### 2.8 In Vivo Safety Evaluation

The HE staining analysis of the sections of main organs of all the treated mice exhibited negligible organ toxicity of all formulations (Figure 6H). Aspartate aminotransferase (AST), alanine aminotransferase (ALT) and glutamyl transpeptidase (GGT) are the markers of inflammatory damage to the liver. Lactate dehydrogenase (LDH) and creatine kinase (CK) are the indicators of inflammatory damage of the heart. Urea (UREA) and creatinine (CREA) are the indicators of inflammatory damage of the kidneys. The hematological analysis showed that the values of AST, ALT, GGT, LDH, CK, UREA, and CREA in the mice treated with FeAMV were all within the normal range (Figure 6I,J), suggesting that the FeAMV were generally safe and would not induce the damage to the heart, liver, and kidneys.

## 3 Conclusion

To overcome the current challenges faced in ferroptosis‐related cancer treatment and to improve the targeting efficiency and tumor penetration of nanocarriers for increased cancer therapy, a supramolecularly engineered conjugate of living bacteria and cell membranes‐coated ferroptosis inducers is developed for the synergistic ferroptosis and immunotherapy of tumors with enhanced specificity and effectiveness. Benefiting from the excellent targeting ability of VNP and CM, FeAMV could specifically accumulate at the tumor sites and release the ferroptosis inducers. The combination of Fe_3_O_4_ and SAS could induce significant ferroptosis, which could effectively inhibit tumor growth and generate immunogenicity. The colonization of VNP could also promote immune responses, which not only improves the intracellular H_2_O_2_ level, but also promotes the adaptive immune responses, resulting in improved ferroptosis and immunotherapy. Consequently, the supramolecular conjugate of Fe_3_O_4_ and VNP could mutually magnify the ferroptosis and immunotherapy, resulting in the effective therapy of tumors. This work not only provides a novel supramolecular method for engineering bacteria as stable, targeted drug carriers, but also offers an insight into therapy of tumors with enhanced specificity and efficacy.

## 4 Experimental Section

### Animal Ethical Statement

All animal procedures were performed in accordance with the Guidelines for Care and Use of Laboratory Animals of University of Macau and approved by the Animal Ethics Committee of University of Macau (UMARE‐017‐2020).

### Statistical Analysis

Statistical analysis relied on an un‐paired two‐tailed *t*‐test methods using GraphPad Prism 9.0. Statistical significance was annotated with ^*^ *p* < 0.05, ^**^ *p* < 0.01, ^***^ *p* < 0.001, ^****^ *p* < 0.0001, respectively. All data were presented as the mean value ± standard (*n* = 3 or 5) deviation of independent runs.

## Conflict of Interest

The authors declare no conflict of interest.

## Author Contributions

B.X., H.Z., and Y.‐F.D. contributed equally to this work. Beibei Xie: Conceptualization, Investigation, Data curation, Writing‐original draft. Huichao Zhao: Investigtion, Data curation, Writing‐original draft. Yuan‐Fu Ding: Investigation, Data curation, Formal analysis, Methodology. Ziyi Wang: Investigation, Methodology. Cheng Gao: Formal analysis, Methodology. Shengke Li: Methodology. Kehan Zhang: Methodology. Huazhong Yu: Data curation, Visualization. Seong Wan Ip: Investigation. Ruibing Wang: Conceptualization, Resources, Supervision, Writing‐review & editing.

## Supporting information

Supporting Information

## Data Availability

The data that support the findings of this study are available from the corresponding author upon reasonable request.
