# Supplementary material for: Supramolecularly Engineered Conjugate of Bacteria and Cell Membrane‐Coated Magnetic Nanoparticles for Enhanced Ferroptosis and Immunotherapy of Tumors
Source: Adv Sci (Weinh). 2023 Oct 18;10(34):2304407. doi: 10.1002/advs.202304407 (PMC10700203; doi:10.1002/advs.202304407)
Supplement: Supplementary file 1 — Supporting Information [file ADVS-10-2304407-s001.pdf]

ADVANCED SCIENCE
Open Access

Supporting Information

for *Adv. Sci.*, DOI 10.1002/advs.202304407

Supramolecularly Engineered Conjugate of Bacteria and Cell Membrane-Coated Magnetic Nanoparticles for Enhanced Ferroptosis and Immunotherapy of Tumors

*Beibei Xie, Huichao Zhao, Yuan-Fu Ding, Ziyi Wang, Cheng Gao, Shengke Li, Kehan Zhang, Seong Wan Ip, Huazhong Yu and Ruibing Wang**

Supramolecular Conjugate of Living Bacteria and Cell Membrane-Coated Magnetic Nanoparticle for Enhanced Ferroptosis and Immunotherapy of Tumors

Beibei Xie, Huichao Zhao, Yuan-Fu Ding, Ziyi Wang, Cheng Gao, Shengke Li, Kehan Zhang, Seong Wan Ip, Huazhong Yu, and Ruibing Wang*

Materials

$FeCl_3 \cdot 6H_2O$, sodium acetate, ethylenediamine, and SAS were obtained from Shanghai Aladdin Biochemical Technology Co., Ltd. DSPE-PEG(2000)-NHS, DSPE-PEG(2000)-SH, DSPE-PEG(2000)-ADA were bought from Xi'an Ruixi Biological Technology Co., Ltd. RPMI 1640 medium, Fetal Bovine Serum (FBS), Trypsin-EDTA, and Trypsin were purchased from HyClone/Thermo fisher (Beijing, China). Necrostatin-1 (NEC), ferrostatin-1 (Fer-1), iron chelator deferoxamine (DFO), GSH Assay Kit, Hoechst 33342, autophagy inhibitor 3-MA, apoptosis inhibitor carbobenzoxy-valyl-alanyl-aspartyl-[O-methyl]-fluoromethylketone (FMK) and DCFH-DA were purchased from Beyotime Biotechnology Inc. (Shanghai, P. R. China). Anti-mouse CD11c antibody, anti-mouse CD206 antibody, anti-mouse CD80 antibody, and anti-mouse CD86 antibody were gotten from BioLegend, Inc. Lipopolysaccharide (LPS) was obtained from Sigma Aldrich (USA). CB[7] was synthesized in our laboratory. All the chemicals were analytic grades and used without further purification. Attenuated VNP was bought from the American Type Culture Collection.

Methods

Synthesis of DSPE-PEG-CB[7]

DSPE-PEG-CB[7] was synthesized refer to our previously reported method through the click reaction between monoallyloxy CB[7] and DSPE-PEG-SH [1]. Briefly, DSPE-PEG-SH (1 mM) was mixed with monoallyloxy CB[7] (1 mM) and dissolved in deionized water, subsequently irradiated by UV light at room temperature for 24 h. After the dialysis purification (M_W = 2000 Da), the DSPE-PEG-CB[7] was obtained.

Preparation of VNP-CB[7]

VNP was planted on the LB agar plate and inoculated overnight at 37°C. Afterward, the VNP colony was picked, diluted into a 5 mL culture medium, and grown overnight to reach an OD600 of about 0.4. Then VNP was washed with PBS by centrifugation (2300 *g*, 3 min) and re-suspended in PBS with 100 μM of DSPE-PEG-CB[7] for 6 h. The VNP-CB[7] was obtained after centrifugation and washing with PBS, and the pellet was suspended with PBS at OD600 of about 0.4 for further use.

Preparation of Fe_3O_4 nanoparticles

Fe_3O_4 nanoparticles were prepared through a previously published method [2]. Briefly, $FeCl_3 \cdot 6H_2O$ (1 g) was dissolved in 20 mL of EG to generate a uniform solution, then 3 g of NaAC and ETH (10 mL) were added in the above solution and stirred for 30 min. The mixed solution was sealed in a Teflon-lined stainless-steel autoclave and heated for 8 h at 200 °C. After the autoclave was cooled to room temperature, the dark brown products were alternately washed with ethanol and deionized water and separated by a magnet during each step. The resulting Fe_3O_4 nanoparticles were suspended in deionized water for further use.

The loading of SAS in Fe_3O_4 nanoparticles

To load the SAS into the pores of mesoporous Fe_3O_4 nanoparticles, 50 mg SAS was dissolved in 10 mL DMSO to obtain a stock SAS solution with a concentration of 5 mg/mL. 5 mg Fe_3O_4 nanoparticles were mixed with 0.5 mL SAS solution (5 mg/mL) and stirred overnight. The FeA was obtained through centrifugation and washing with ethanol and deionized water. The loading amount of SAS was determined through the UV spectrum of SAS.

Preparation of CM-ADA

Briefly, 4T1 cells were first cultured with RPMI 1640 culture medium containing 10% FBS and 1% antibiotics overnight, and then 20 μM DSPE-PEG-ADA was added for incubation for 1.5 h in a 37°C incubator with 5% CO_2. 4T1 cells were harvested and washed with PBS 3 times. Subsequently, 4T1 cells were suspended with cold TM buffer (0.01M Tris and 0.001M $MgCl_2$) and placed on ice for 1-2 hours. Later, the suspension was extruded 20 times to lyse cells. 1M of sucrose solution and one-third of the TM buffer volume were added to dilute the solutions and obtain the final concentration of 0.25 M. Then, the solution was centrifuged (3000 *g*, 4°C, 10 min) to remove the pellet mainly containing the cell nucleus. The supernatant was collected for further centrifugation with a speed of 3000 g for 30 min at 4°C. The obtained precipitate was the CM-ADA, which was then dispersed with PBS buffer and stored at -80°C for further use.

Preparation of FeAM

The suspension was mixed with CM-ADA suspension at a mass ratio of around 1:1 and sonicated for 3 min. The FeAM was obtained through centrifugation and washing with PBS.

Preparation of FeAMV

1mL FeAM (100 μg/mL) was mixed with VNP-CB[7] (10^6 CFU) in PBS buffer and incubated at 37 °C for 30 min. The FeAMV was gained through the host-guest interaction between ADA and CB[7]. Later, the mixture was washed, centrifuged, and resuspended in PBS buffer for further use.

Nanoparticle characterization

SU-8010 (Hitachi, Japan) under the operating voltage of 3 kV was applied for SEM characterization. JEM-1011 TEM (JEOL, Japan) with an accelerating voltage of 100

kV was used for TEM imaging. The TEM species were prepared by dropping 5 μL of suspensions (0.05 mg/mL) on the copper grid and then air drying. The hydrodynamic diameters and zeta potential of samples were analyzed on the DLS (Malvern Zetasizer Nano ZS). The iron content was determined on the Thermo iCAP Qs ICP-MS.

Membrane protein characterization

The membrane protein of FeM was characterized via sodium dodecyl sulfate-polyacrylamide gel electrophoresis (SDS-PAGE). Briefly, extracted CM-ADA and FeM nanoparticles were lysed with RIPA lysate, then the total protein concentration was decided by BCA Protein Assay Kit (Beyotime, China). All samples were mixed with SDS-PAGE sample loading buffer (Beyotime, China), and heated at 100 °C for 10 min. Then, 40 g/well of each sample was run on a 10% SDS-polyacrylamide gel at 80 V, lasting for 30 min first, followed by 120 V for 1 h. Later, the gel was dyed with Coomassie Blue for 20 min, washed with deionized water, and eventually imaged.

Drug release behavior

To analyze the drug release behavior of SAS, 2 mg of FeAM were suspended in 2 mL of PBS buffer solution at different pH of 7.4, 6.5, and 5.5, respectively, to yield 1 mg/mL suspension, and the suspensions were transferred into dialysis bags (M_D=1000 Da). The dialysis bags were soaked in 20 mL of release medium (buffer with different pH values), respectively, and stirred gently at room temperature. At different time points, including 0, 0.25, 0.5, 1, 2, 3, 4, 6, 8, 10, 12, and 24 h, 1 mL of release solution was collected for absorption measurement, and 1 mL fresh buffer solution at different pH values was supplemented to keep release medium volume constant. The drug release behavior of bare FeA in the pH 7.4 PBS buffer (0.02 M) was used as a control. SAS has a characteristic absorption peak at 359 nm, and the absorbance of SAS at 359 nm was linear with SAS concentration. The release amount of SAS in the dialysis bag at each time point was measured by DR600 UV-Vis Spectrophotometer. Each experiment was performed in triplicate. The encapsulation efficiency of SAS in the nanoparticles were measured by a DR600 UV-Vis spectrophotometer.

***In vitro* biocompatibility evaluation**

MTT assay was used to assess the biocompatibility of nanoparticles against LO2 cells. First, LO2 cells were inoculated in a 96-well plate with a density of 10^4 cells/well and cultured overnight. Then, Fe_3O_4, FeM, and FeMV with different concentrations of Fe_3O_4 (0, 12.5, 25, 50, and 100 μg/mL) were added and treated for 24 h. Later, the culture medium was discarded, and 100 μL culture medium involving MTT (5 mg/mL) was applied to each well, subsequently incubated for another 4 h. Then the medium was discarded, followed by adding 100 μL DMSO to dissolve formazan. Eventually, the cytotoxicity of nanoparticles to LO2 cells was assessed through a Microplate reader.

***In vitro* cytotoxicity assay**

Similarly, an MTT assay was applied to investigate the cytotoxicity of different

formulations against 4T1 cells. First, 4T1 cells were inoculated in a 96-well plate with a density of 10^4 cells/well and incubated overnight. When cells reached around 70% confluence, cells were incubated with PBS, VNP, SAS, Fe_3O_4, FeA, FeAM, FeMV, and FeAMV containing the same concentration of SAS (20 μg/mL), Fe_3O_4 (100 μg/mL) and VNP (10^6 CFU). After treated for 24 h, the culture medium was removed, and the FBS-free medium involving MTT (5 mg/mL) was added and incubated for another 4 h. Then the medium was discarded, followed by adding 100 μL DMSO to each well to dissolve formazan. Eventually, the cytotoxicity of different groups against 4T1 cells was assessed by a Microplate reader at 490 nm.

Evaluation of ferroptosis

To explore the pattern of FeAMV-induced cell death, 3-MA (autophagy inhibitor), apoptosis inhibitor z-VAD-FMK, NEC (necroptosis inhibitor), DFO (iron chelator deferoxamine), and Fer-1 (ferroptosis inhibitor) were applied in the cytotoxicity assay. Briefly, 4T1 cells were inoculated in the 96-well plate and incubated overnight. When reaching 70-80% confluence, the cells were treated with FeAMV (100 μg/mL) with 3-MA (10 μM), z-VAD-FMK (10 μM), NEC (10 μM), DFO (100 μM), and Fer-1 (10 μM) alternatively for 24 h. After that, cell viability was measured by MTT assay.

Intracellular ROS measurement

DCFH-DA, the ROS probe, was applied to investigate intracellular ROS production in FeAMV-treated 4T1 cells. Firstly, 1.0×10^5 4T1 cells were first inoculated in the confocal petri dishes (20 mm) and cultured in 1.0 mL RPMI 1640 medium with 10% FBS and overnight. Subsequently, FeAMV (50 μg/mL, 100 μg/mL, or 200 μg/mL) with or without iron chelator (DFO) or ferroptosis inhibitor (Fer-1) was added. Following 24 h of incubation, Hoechst 33342 and DCFH-DA were added to stain the cell nucleus and intracellular ROS. Confocal images were captured using a CLSM (Zeiss LSM710).

Intracellular GSH level measurement

The intracellular GSH level was measured in FeAMV-treated 4T1 cells by using the GSH assay kit (Beyotime, China). 4T1 cells were inoculated with a density of 2×10^5 each well in the 6-well plate. Then PBS, SAS, Fe_3O_4, VNP, FeA, FeMV, and FeAMV with an equivalent concentration of SAS (20 μg/mL), Fe_3O_4 (100 μg/mL), or VNP (10^6 CFU) were added to the 4T1 cells culture. After incubation for 12 h, the cells were treated with trypsin to collect cell suspension; then, the suspension was washed with PBS through centrifugation. The supernatant was discarded and treated with a 3-fold cell volume of protein removal reagent S solution. The sample was rapidly freeze-thawed twice using liquid nitrogen and a 37°C water bath. The sample was then placed in the ice bath for 5 min and centrifuged (10,000 *g*) at 4°C for 10 min. Last, the supernatant was used to determine total GSH by the GSH assay kit.

***In vitro* DCs maturation experiment**

Bone marrow-derived iDCs were harvested from BALB/c mice aged 6 to 8 weeks following the instruction reported before [3]. The 24-well transwell system with a 0.4

μm polycarbonate porous membrane was used to study *in vitro* DCs maturation experiment. First, 2×10^4 4T1 cells per well were inoculated in the upper well. Subsequently, cells were incubated with PBS, SAS, Fe_3O_4, VNP, FeA, FeAM, and FeAMV with an equivalent concentration of SAS (20 μg/mL), Fe_3O_4 (100 μg/mL), or VNP (10^6 CFU) for 24 h. After that, the upper well was transferred and co-incubated with DCs previously inoculated in the bottom well. After co-culture for 24 h, DCs in the bottom were harvested and stained with anti-CD11c antibody and anti-CD86 antibody for 1 h. Later, DCs were resuspended by PBS and assessed by flow cytometry after washing by centrifugation.

In vitro **immune analysis**

The TNF-α and IL-6 levels were evaluated using ELISA kits (Lai Er Bio-Tech, China) in a 24-well transwell system with a 0.4 μm polycarbonate porous membrane. First, 4T1 cells were inoculated in the upper well. When the cells reached 70-80% confluence, PBS, SAS, Fe_3O_4, VNP, FeA, FeAM, and FeAMV with an equivalent concentration of SAS (20 μg/mL), Fe_3O_4 (100 μg/mL), or VNP (10^6 CFU) were added to the cells and incubated for 24 h. Afterward, the upper wells were transferred and co-incubated with Raw 264.7 previously inoculated in the bottom well. After the co-culture, the supernatant was obtianed for cytokine TNF-α and IL-6 analysis by ELISA assay kits.

In vivo **bio-distribution study**

Female BALB/c mice (6 to 8 weeks) were obtained from the Animal Center of the University of Macau. 10^6 4T1 cells were injected to the hind legs of mice and observed for 12 days. Then, the mice were separated into 5 groups at random (n=3), and the free Cy5.5, Fe_3O_4, VNP, FeAM, and FeAMV were intravenously injected into the mice through the tail vein (2 mg/kg Cy5.5). At predetermined time points after injection (0, 6, 12, 24, 36, and 48 h), mice were anesthetized, and an *in vivo* imaging system (IVIS) was applied to capture fluorescence signals. All mice were sacrificed 48 hours later to collect the organs and tumor for fluorescence imaging.

Later, 0.02 mg tissue was cut from the organs or tumors for grinding to observe the distribution of Fe in different organs. The ground mixture was then soaked in aqua regia overnight, diluted with a 2% nitric acid solution, and detected through the Thermo iCAP Qs ICP-MS to measure Fe content.

Similarly, 0.02 mg tissue was cut from the organs or tumors for grinding to observe the distribution of VNP in different organs. Single-cell suspension was obtained using a Falcon® 40 μm cell strainer. After centrifuging (300 *g*, 5 min), the supernatant was diluted at different times for plating and colony counting. Later, 100 μL bacteria solution was added to the agar plate, spread uniformly, and incubated at 37°C overnight for colony counting.

In vivo **anti-tumor study**

Female BALB/c mice (6 to 8 weeks) were obtained from the Animal Center of the University of Macau. 10^6 4T1 cells were injected to the hind legs to get 4T1 tumor-bearing mice. Once the tumor volume reached about 80 mm^3, the mice were

separated into 7 groups at random and intravenously injected with PBS, SAS, VNP, FeA, FeAM, FeMV and FeAMV via the tail vein with an equivalent SAS concentration of 5 mg/kg on day 1, 3, 5, 7, 9. The tumor volume and mice weight were recorded every 3 days until the test ended. The tumor volume was calculated as the following formula: $V = (L \times W^2)/2$, where L and W denoted the maximum and minimum diameters. When the 14-day treatment ended, the mice were sacrificed to harvest their major organs, tumors, and blood for H&E and TUNEL staining, Fe and VNP distribution evaluation, the study of M1/M2 macrophage ratio in tumor section, and IL-6 and TNF-α expression level in the tumor site.

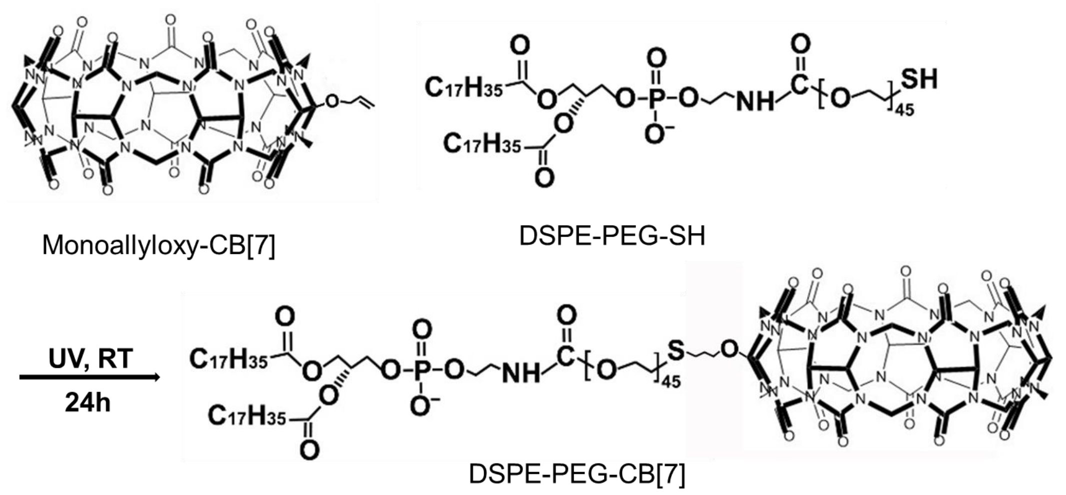

Figure S1. Synthetic route of DSPE-PEG-CB[7].

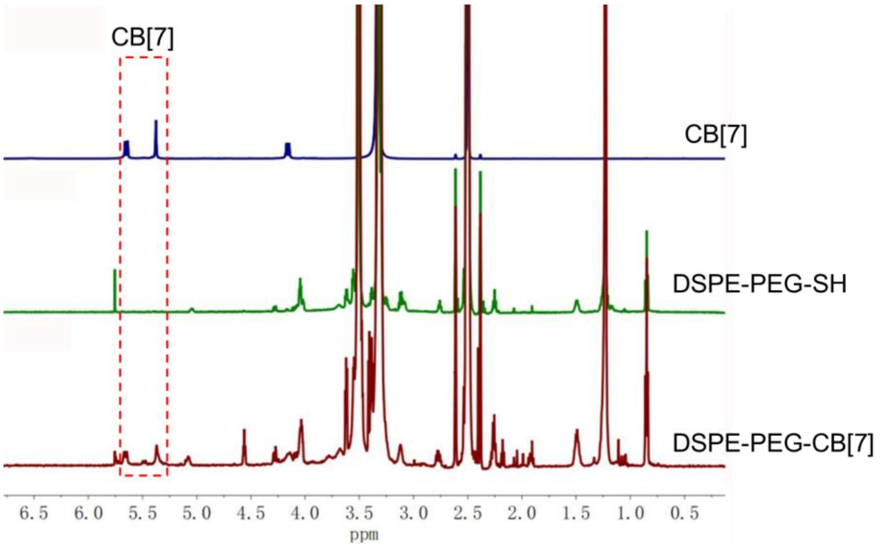

Figure S2. ^{1}H NMR spectrum of CB[7], DSPE-PEG-SH, and DSPE-PEG-CB[7].

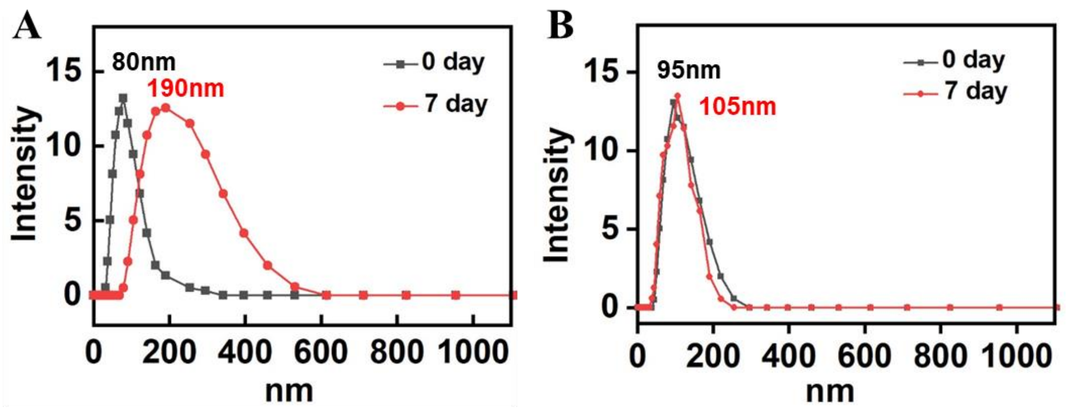

Figure S3. Size distribution of A) Fe_3O_4 nanoparticles and B) FeM on Day 0 and Day 7, respectively.

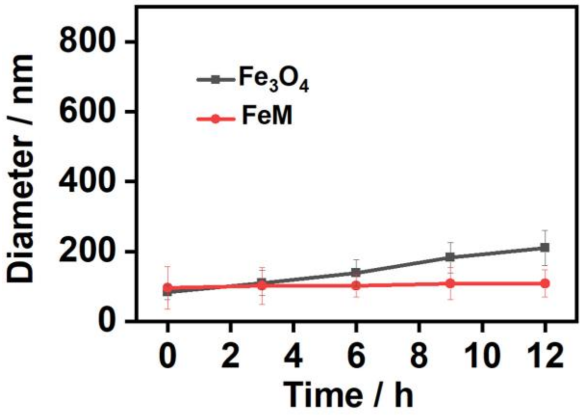

Figure S4. The mean hydrodynamic diameters of Fe_3O_4 and FeM after incubation in 10% FBS for different durations determined by DLS.

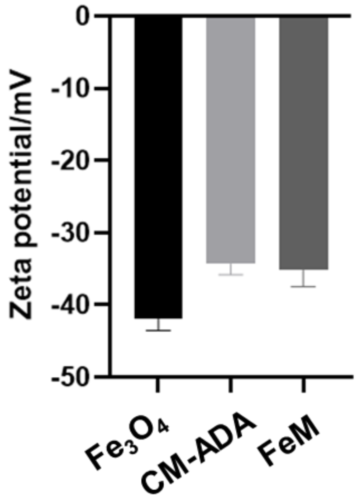

Figure S5. The Zeta potential of Fe_3O_4, CM-ADA, and FeM.

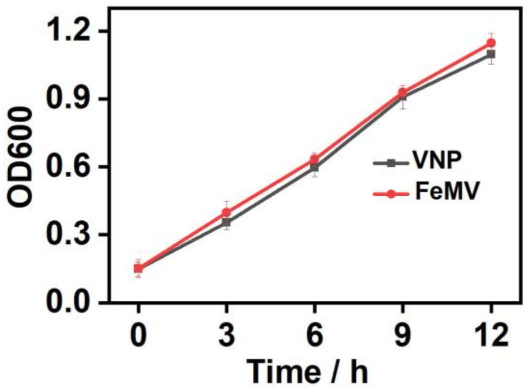

Figure S6. The proliferation profiles of VNP and FeMV for up to 12 h.

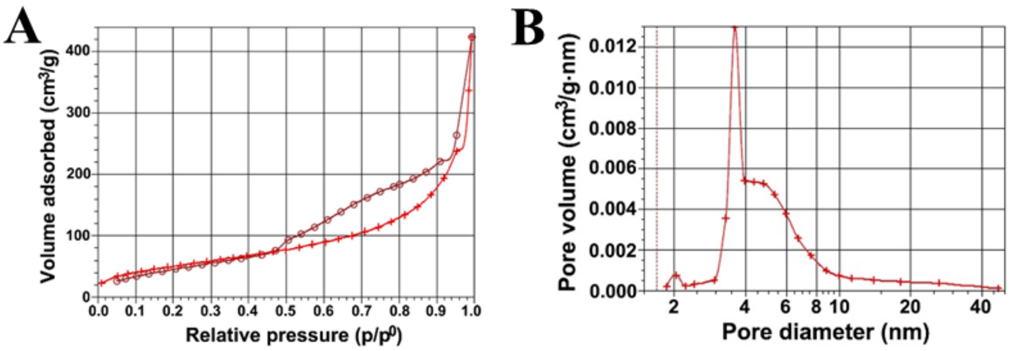

Figure S7. A) N_2 absorption-desorption isotherms and **B)** pore size distribution of Fe_3O_4 nanoparticles.

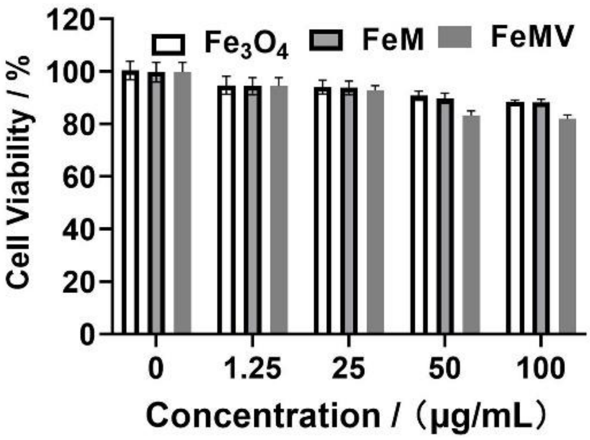

Figure S8. In vitro cytotoxicity of Fe_3O_4 nanoparticles, FeM, and FeMV with different concentrations of Fe_3O_4 against LO2 cells after 24 h incubation.

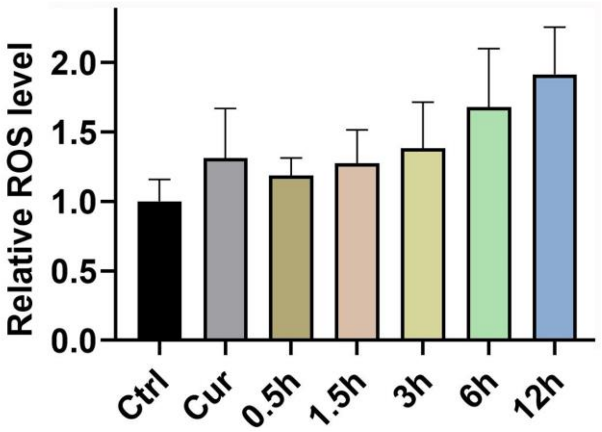

Figure S9. The generation of ROS in Raw264.7 cells incubated with VNP (10^6 CFU) at different time.

References

1. C. Gao, et al., Supramolecular Macrophage-Liposome Marriage for Cell-Hitchhiking Delivery and Immunotherapy of Acute Pneumonia and Melanoma, Adv. Funct. Mater. 31 (2021) 2102440.

2. M. Chu, et al., Near-infrared laser light mediated cancer therapy by photothermal effect of Fe3O4 magnetic nanoparticles, Biomaterials 34 (2013) 4078-4088.

3. L. Liu, et al., An optimized method for the induction and purification of mouse bone marrow dendritic cells, J Immunol Methods 495 (2021) 113073.
